# Cinema to Help a Hospital

**Published:** 1920-09-25

**Authors:** 


### Cinema to Help a Hospital.

Whatever may be the future of hospitals—whether they are continued on the voluntary principle, as at present, or are State-aided—Hove is to make a big effort to put its hospital on a sound financial basis. Despite its population bordering on the status of a county borough, Hove has only one hospital that takes in accidents; and where treatment is wholly free, and bearing in mind its hundreds of wealthy residents—it is the West End of Brighton—it does not redound to its credit that its only hospital finds it difficult to carry on. A civic appeal is now being made, coupled with a forthcoming Oriental bazaar organised by the Mayor and Mayoress (Councillor and Mrs. F. W. A. Cushman), and in connection with the effort there are to be cinema pictures of the hospital and its work. They are to be shown locally at first, but it may be hoped they will go much further afield. The rapid growth of picture palaces show that they are a big draw on the general public, and probably have been to the detriment of the hospitals in the sense that stray coppers that used to find their way to the hospital box in the home are now handed over to the cinema. Films depicting something of the work of the hospital, how it is hindered for the want of the necessary wherewithal, and a little of its pathetic and, perhaps, its gruesome side, should be the means of bringing home to the habitués of the picture theatres their duty towards the institution which first comes to their mind in time of stress. It is satisfactory to hear that the film is coming to the rescue of a hospital; but considerable care will have to be exercised in its making for the cinema houses.

